# Treatment of Disease by Faith and Prayer, Instead of Medicine

**Published:** 1868-06

**Authors:** 


### Treatment of Disease by Faith and Prayer, instead of Medicine.

In the current number of *Good Words*, is an account by Mr. William Gilbert, of visits paid by him to two of the Continental establishments at which "the treatment of disease by faith and prayer instead of medicine" is carried out systematically.

The institution first visited was that of the Lutheran pastor, Christoph Blumhardt, in the Black Forest; and here Mr. Gilbert found a handsome house, with gardens, etc.—in fact a bankrupt water-cure establishment converted into a semi-religious house, with the gambling and dancing *salon* turned into a chapel. Here were assembled just that class of valetudinarians who might be met with at any fashionable but quiet resort of invalids, averaging a hundred and sixty in number, who promenaded, attended chapel, took regular meals, and kept early hours, and went away benefited accordingly. A conversation with the pastor elicited that "he did not deny the efficacy of medicine, but at the same time had a much greater reliance on the efficacy of prayer, being fully convinced from his own experience, which was great, that the majority of diseases could be cured by prayer and faith, without the application of scientific remedies. In surgical cases he did not deny the necessity of calling in a skillful surgeon." Our surgical brethren will feel complimented at this admission, which is not in strict accordance with the views of one of the "peculiar" witnesses at the recent inquest. Mr. Gilbert did not see any cases of cure, nor were there in the institution any persons suffering from serious diseases; he was assured, however, that there had been "some wonderful cases" during the preceding months.

Dorotha Trudel was a young woman who resided at Männedorf, on the banks of Lake Zurich, and who, being of a pious disposition, betook herself to prayer when some members of her family had been given over by the physicians. They recovered, and on sickness again breaking out in the village Dorotha was called in to pray over the sick, who recovered without any medical advice. Unfortunately, medical jealousy appears to have been excited by the reputation she thus obtained, and a prosecution followed, with the imposition of a fine, which, however, was remitted on appeal. Persecution naturally gave publicity, and publicity only increased reputation, so that Dorotha became the rage, and sick folk flocked together from all sides. In the midst of her successes, and in spite of them, Dorotha unfortunately died, (in 1862,) and her "system" is now carried on by others, with what success we are not informed.—*Lancet*, March 7, 1868.

